# Follow-up at 1 year and beyond of women with gestational diabetes treated with insulin and/or oral glucose-lowering agents: a core outcome set using a Delphi survey

**DOI:** 10.1007/s00125-019-4935-9

**Published:** 2019-07-04

**Authors:** Delia Bogdanet, Catriona Reddin, Esther Macken, Tomas P. Griffin, Narjes Fhelelboom, Linda Biesty, Shakila Thangaratinam, Eugene Dempsey, Caroline Crowther, Sander Galjaard, Michael Maresh, Mary R. Loeken, Angela Napoli, Eleni Anastasiou, Eoin Noctor, Harold W. de Valk, Mireille N. M. van Poppel, Andrea Agostini, Cheril Clarson, Aoife M. Egan, Paula M. O’Shea, Declan Devane, Fidelma P. Dunne

**Affiliations:** 1 College of Medicine, Nursing and Health Sciences, National University Ireland, University Road, Galway, H91 TK33 Ireland; 2 Queen Mary, University of London, Women’s Health Research Unit, London, UK; 3 INFANT Centre and Department of Paediatrics & Child Health, University College Cork, Cork, Ireland; 4 Liggins Institute, The University of Auckland, Auckland, New Zealand; 5 Department of Obstetrics and Gynaecology, Division of Obstetrics and Prenatal Medicine, Erasmus MC, University Medical Centre Rotterdam, Gravendijkwal 230, 3015 CE Rotterdam, the Netherlands; 6 Department of Obstetrics, St Mary’s Hospital, Manchester University Hospitals NHS Foundation Trust, Manchester Academic Health Science Centre, Manchester, UK; 7 Section of Islet Cell and Regenerative Biology, Joslin Diabetes Center, Boston, MA USA; 8 Department of Medicine, Harvard Medical School, Boston, MA USA; 9 Department of Clinical and Molecular Medicine, Sant’Andrea University Hospital, Sapienza, University of Rome, Rome, Italy; 10 Department of Endocrinology, Metabolism and Diabetes Centre, Alexandra Hospital, Athens, Greece; 11 Department of Endocrinology, University Hospital Limerick, Limerick, Ireland; 12 Department of Internal Medicine, University Medical Centre Utrecht, Utrecht, the Netherlands; 13 Institute of Sport Science, University of Graz, Graz, Austria; 14 A.S.L Viterbo Distretto A, Consultorio Montefiascone, Rome, Italy; 15 Department of Pediatrics, University of Western Ontario, London, ON Canada; 16 Lawson Health Research Institute, London, ON Canada; 17 Division of Endocrinology, Diabetes and Metabolism, Mayo Clinic, Rochester, MN USA

**Keywords:** Core outcome set, Gestational diabetes mellitus, Insulin, Oral hypoglycaemic agents

## Abstract

**Aims/hypothesis:**

Gestational diabetes mellitus (GDM) is linked with a higher lifetime risk for the development of impaired fasting glucose, impaired glucose tolerance, type 2 diabetes, the metabolic syndrome, cardiovascular disease, postpartum depression and tumours. Despite this, there is no consistency in the long-term follow-up of women with a previous diagnosis of GDM. Further, the outcomes selected and reported in the research involving this population are heterogeneous and lack standardisation. This amplifies the risk of reporting bias and diminishes the likelihood of significant comparisons between studies. The aim of this study is to develop a core outcome set (COS) for RCTs and other studies evaluating the long-term follow-up at 1 year and beyond of women with previous GDM treated with insulin and/oral glucose-lowering agents.

**Methods:**

The study consisted of three work packages: (1) a systematic review of the outcomes reported in previous RCTs of the follow-up at 1 year and beyond of women with GDM treated with insulin and/or oral glucose-lowering agents; (2) a three-round online Delphi survey with key stakeholders to prioritise these outcomes; and (3) a consensus meeting where the final COS was decided.

**Results:**

Of 3344 abstracts identified and evaluated, 62 papers were retrieved and 25/62 papers were included in this review. A total of 121 outcomes were identified and included in the Delphi survey. Delphi round 1 was emailed to 835 participants and 288 (34.5%) responded. In round 2, 190 of 288 (65.9%) participants responded and in round 3, 165 of 190 (86.8%) participants responded. In total, nine outcomes were selected and agreed for inclusion in the final COS: assessment of glycaemic status; diagnosis of type 2 diabetes since the index pregnancy; number of pregnancies since the index pregnancy; number of pregnancies with a diagnosis of GDM since the index pregnancy; diagnosis of prediabetes since the index pregnancy; BMI; post-pregnancy weight retention; resting blood pressure; and breastfeeding.

**Conclusions/interpretation:**

This study identified a COS that will help bring consistency and uniformity to outcome selection and reporting in clinical trials and other studies involving the follow-up at 1 year and beyond of women diagnosed with GDM treated with insulin and/or oral glucose-lowering agents during pregnancy.

**Electronic supplementary material:**

The online version of this article (10.1007/s00125-019-4935-9) contains peer-reviewed but unedited supplementary material, which is available to authorised users.

## Introduction

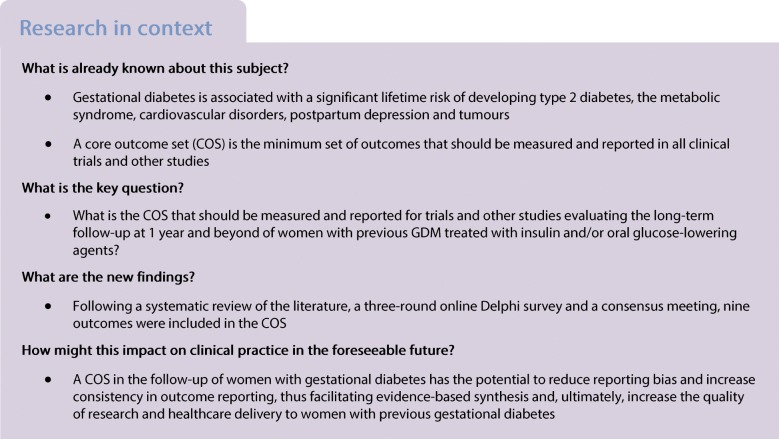



The prevalence of gestational diabetes mellitus (GDM) is increasing worldwide and ranges between 5.2% and 40.4% across countries; this wide variability reflects multiple factors such as BMI, ethnicity, country income, and also the diagnostic criteria used [1]. Based on the International Association of Diabetes in Pregnancy Study Group (IADPSG) criteria, the European prevalence of GDM is approximatively 13% [2, 3]. Medical nutritional therapy (diet and exercise) is the first step in the treatment of GDM [4]. However, if the desired glycaemic goals are not achieved, pharmacological treatment will be required.

GDM is linked with a substantial lifetime risk of developing type 2 diabetes. A meta-analysis conducted on studies published over the last 50 years showed that women with a history of GDM have a higher risk of developing type 2 diabetes (RR 7.4) compared with women with normal glucose tolerance (NGT) in pregnancy [5]. Women with previous GDM are more likely to develop the metabolic syndrome [6] and cardiovascular disorders [7] in later life. Several studies have shown that women with GDM have a higher risk of developing postpartum depression [8, 9]. There is also a growing body of literature associating a history of GDM with the development of tumours, particularly breast and endometrial tumours [10].

Women with GDM should be screened for persistent diabetes or impaired fasting glucose and/or impaired glucose tolerance at 6–12 weeks postpartum using non-pregnancy criteria [11]. However, there is no standardised approach to the long-term follow-up of women with a previous GDM diagnosis. The results of clinical trials evaluating comparable interventions are usually summarised in systematic reviews and meta-analyses that provide the basis for guidelines and treatment recommendations. However, there is little consistency in outcome selection and reporting in clinical trials involving this population. This inconsistency raises concern for possible outcome selection bias, makes significant research synthesis difficult and limits the ability to combine the findings of individual studies into summary estimates. One way to overcome this is to develop a core outcome set (COS).

A COS is the minimum set of outcomes that should be consistently measured and reported in all clinical trials (and other studies). However, this does not restrict researchers from adding additional outcomes. A minimum set of outcomes will provide greater uniformity of reporting in clinical trials and more data to impact meta-analyses. Also, a COS will reduce study heterogeneity and the risk of reporting bias by consistently measuring and reporting these outcomes.

The Core Outcome Measures in Effectiveness Trials (COMET) initiative aims to standardise outcome reporting in trials, facilitates participation of diverse experts undertaking research and minimises duplication of work [12, 13]. The Core Outcome Set STAndards for Reporting (COS-STAR) is a checklist designed to be applicable regardless of the consensus methods used to develop the COS and the various participant groups [14]. The COS-STAR checklist provides guidance for minimal COS study reporting and its purpose is to promote the transparency and completeness of reporting in all COS studies. The CoRe Outcomes in Women’s and Newborn health (CROWN) initiative encourages the development of COSs in women’s and newborns’ health.

The aim of this study was to develop a COS for trials and other studies evaluating the long-term follow-up at 1 year and beyond of women with previous GDM treated with insulin and/oral glucose-lowering agents. This study focuses only on women with GDM treated with insulin and oral glucose-lowering agents as this population has more severe glucose abnormalities and are more likely to progress to type 2 diabetes, obesity and the metabolic syndrome [15, 16].

## Methods

This study is registered in the COMET database [17]. Ethical approval for the study was obtained from the Galway University Hospitals Research Ethics Committee (reference CA 1905).

The three work packages of the study were: (1) a systematic review of literature that identified all the outcomes reported in clinical trials that involved the long-term follow-up of this population; (2) a Delphi survey in which all outcomes were scored and prioritised by key stakeholder groups to provide a preliminary list of final outcomes; and (3) a consensus meeting where the final list of outcomes was decided.

### Systematic review

Using a broad-based search strategy, the following databases were searched for relevant studies between October 2017 and February 2018: Cochrane Central Register of Controlled Trials (CENTRAL), the Cumulative Index to Nursing and Allied Health Literature (CINAHL), PubMed, EMBASE and Web of Science. ClinicalTrials.gov was also searched for relevant ongoing trials. The reference lists of all included studies were searched for additional studies not retrieved from the electronic database search. There was no time restriction on the date of publication of the studies. There was no language restriction applied to the search strategy. Only RCTs and RCT follow-up studies were included in the systematic review (an example of the search strategy is presented in the electronic supplementary material [ESM] Methods).

In step 1, all identified study titles were reviewed and ineligible studies excluded (F.P. Dunne and D. Bogdanet). In step 2, the remaining studies were appraised by two reviewers (F.P. Dunne and D. Bogdanet) who independently assessed the titles and abstracts of each study included at this stage. Full texts of studies meeting the inclusion criteria and studies for which there was uncertainty regarding inclusion at the title/abstract screening stage were retrieved and reviewed independently (F.P. Dunne and D. Bogdanet). The same two authors extracted the data independently, reviewed the data together, assessed consensus and ensured that all outcomes were identified. Following review by F.P. Dunne, D. Devane, D. Bogdanet, L. Biesty, A. M. Egan and P. M. O’Shea (the study advisory group [SAG]), extracted outcomes were grouped under the following domains: laboratory tests, clinical conditions, physiological variables, diet and exercise, psychological variables and other.

### Delphi method

We conducted a three-round eDelphi survey [18]. This facilitated international participation. Questionnaires were completed online using SurveyMethods software (www.surveymethods.com, SurveyMethods, Dallas, TX, USA, accessed 9 May 2018). Full details of our methods are given in Bogdanet et al (2019) [19] and are described briefly below.

The stakeholder groups comprised: women with a previous diagnosis of GDM, endocrinologists, diabetes nurses, obstetricians, midwives, paediatricians, neonatologists, general practitioners, practice nurses, dietitians, physiotherapists, researchers with expertise in gestational diabetes, policy makers and others (which included clinicians with expertise in gestational diabetes from specialties other than endocrinology and obstetrics, epidemiologists, clinical biochemists and healthcare assistants).

Invitation emails were sent to societies and individual members of the IADPSG, Diabetes Ireland, Irish Endocrine Society (IES), IDF, International Federation of Gynecology and Obstetrics (FIGO), European Board and College of Obstetrics and Gynaecology (EBCOG), Irish Nutrition and Dietetic Institute (INDI), Association of Clinical Biochemists in Ireland (ACBI), Irish Institute of Obstetricians and Gynaecologists, Saolta Healthcare Group (Ireland), EASD, Diabetic Pregnancy Study Group (DPSG) of the EASD and the Royal College of Physicians Ireland (RCPI) (divisions of Endocrinology, Obstetrics and Endocrinology and Paediatrics). All participants were asked to forward the invitation to others whom they regarded as having the required expertise. Additionally, women with a history of GDM were contacted through their clinic by the authors and by additional study participants and, following consent, were forwarded the survey link or given a printed form of the survey. Women with GDM were from a number of clinics; the final group who participated in the consensus meeting were from the Galway clinic.

Study participants gave informed consent prior to the submission of any answers and the following information was also requested: name, email address, sex, stakeholder group and country of residence. Participants were given information about the study and about COSs. Participants were encouraged to complete the eDelphi questionnaire in each round. An email reminder was sent to anyone who did not respond after 7 and 14 days and also 3 days and 1 day before the end of the round.

In the first round of the survey, all the outcomes identified in the systematic review were presented to the participants grouped by domain. The study participants were asked to rate each outcome on a nine-point Likert scale (1–3 limited importance; 4–6 important but not critical, 7–9 critical). We provided all participants with plain English explanations of the outcomes included in the survey. Participants were invited to suggest additional relevant outcomes (no limit to the number of outcomes suggested) using free-text responses. If two or more study participants nominated an outcome, that outcome was included in round 2 of the survey.

All stakeholder groups were grouped into three broader groups, i.e. clinicians, women with a previous diagnosis of GDM and researchers/policy makers. Descriptive statistics were used to summarise the results from round 1. We sent individual results, the results of each stakeholder group and the results of the total group to each study participant. All outcomes including the additional outcomes suggested in round 1 (by two or more participants) were carried forward to round 2. All respondents to round 1 were invited to participate in round 2 and asked to re-rate the outcomes. All outcomes that scored 7–9 on the Likert scale in ≥70% of answers and 1–3 in <15% of answers were carried forward to round 3. Each participant who completed round 2 was emailed their individual results and the results of each stakeholder group and the total group and was invited to participate in round 3 and re-score retained outcomes. Outcomes were classified as ‘consensus in’ (≥70% participants scoring as 7–9 and <15% scoring as 1–3) or ‘consensus out’ (≥70% scoring as 1–3 and <15% scoring as 7–9). The ‘consensus in’ and borderline outcomes were brought forward to the consensus meeting.

### Consensus meeting

The consensus meeting involved representatives from each stakeholder group. The participants discussed each outcome brought forward from round 3. If necessary, the outcomes were grouped or renamed in order to facilitate dissemination and usefulness. At the end of the discussion, each participant voted ‘outcome in’ or ‘outcome out’ using the app Poll Everywhere (San Francisco, CA, USA, accessed 27 September 2018) on their electronic device, thus concealing their identity. Outcomes that scored over 70% ‘outcome in’, were included in the final COS.

## Results

A total of 3344 titles and abstracts were identified. Following review of the title and/or abstracts, 62 full text papers were retrieved and assessed for eligibility. A further 37 papers were excluded following full text assessment, leaving 25 papers in the review (ESM Table 1). Following data extraction, 121 individual outcomes were identified. Following the SAG meeting, similar outcomes were combined, leaving a final 116 outcomes to be grouped and included in round 1 (ESM Fig. 1).

The first round of the Delphi survey was sent to 835 participants (societies and individual members). At the end of round 1, there were 288 respondents (34.5%) representing 33 countries and five continents (Table 1). A total of 73% of the respondents were female. The distribution of answers throughout the stakeholder groups in each of the three rounds is presented in Table 1. An additional ten outcomes were suggested by two or more study participants and were included in round 2 (ESM Table 2).

**Table 1:** Characteristics of participants in the Delphi online survey

Variable	Round 1, % (*n* = 288)	Round 2, % (*n* = 190)	Round 3, % (*n* = 165)
Sex (female)	73	73.7	72.1
Experience/background			
Diabetes nurse specialist	5.5	6.3	5.5
Endocrinologist	31.2	35.8	36.4
Obstetrician	14.2	15.8	17.6
Paediatrician	1.7	2.1	0.6
Neonatologist	1.7	1.6	1.2
Midwife	5.5	4.7	4.8
General practitioner	3.8	3.1	3
Practice nurse	0.3	0.5	0.6
Dietitian	1.7	1	0.6
Physiotherapist	1	0	0
Other	11.8	11.6	12.1
Woman with previous diagnosis of GDM	14.2	10	10.3
Researcher with expertise in diabetes	5.5	5.8	6.1
Policy maker	1.4	1.6	0.6
Clinician	78.9	82.6	83.3
Health service user	14.2	10	10.3
Researcher/policy maker	6.9	7.4	6.7
Participant’s country of residence			
Albania	0.3	0.5	0.6
Argentina	7.3	6.8	6.7
Australia	3.1	4.2	3.6
Austria	0.7	1.1	1.2
Belgium	1	0.5	0.6
Canada	1	1.6	1.8
Chile	0.3	0.5	0.6
Croatia	0.7	1.1	1.2
Czech Republic	0.3	0	0
Denmark	3.8	4.2	4.8
France	1.3	2.1	2.4
Germany	1	0.5	0.6
Greece	0.7	1.1	0.6
Hungary	0.3	0	0
India	0.7	0.5	0.6
Ireland	49.8	47.4	49
Israel	0.7	0	0
Italy	2.1	2.6	2.4
Jamaica	0.3	0.5	0.6
Lithuania	1.4	1.6	1.8
Malta	0.3	0.5	0.6
The Netherlands	0.7	1.1	0.6
New Zealand	3.8	4.2	3
Nigeria	0.3	0.5	0.6
Poland	0.7	0	0
Portugal	0.3	0.5	0
Romania	0.7	1.1	1.2
Spain	2.1	1.1	1.2
Sweden	1	1.1	0.6
UAE	0.7	0.5	0.6
UK	7.4	6.4	4.8
USA	4.2	5.8	6.8
Uruguay	0.3	0.5	0

UAE, United Arab Emirates

Round 2 participants were asked to rate 126 outcomes grouped as described in the Methods section. Round 2 was completed by 65.9% of the round 1 responders (190 participants). Similar to round 1, there was a female predominance among responders (73.7%). The distribution of answers amid stakeholder groups was similar to round 1 (Table 1) (clinicians 82.6%, women with a history of GDM 10%, researchers/policy makers 7.4%). All outcomes that scored 7–9 on Likert scale in ≥70% and 1–3 in <15% by study participants were brought forward to round 3 (*n* = 34). The percentage of participants who voted 1–3, 4–6 or 7–9 on each outcome at the end of round 2 is presented in Table 2.

**Table 2:** Percentage of round 2 participants (*n* = 190) scoring each outcome as 1–3, 4–6 or 7–9 on the 9-point Likert scale

Variable	Score 1–3, %	Score 4–6, %	Score 7–9, %
Laboratory tests			
75 g OGTT	5	11	84
Glucose level at 30 min during the 75 g OGTT	53	28	19
Glucose level at 1 h during the 75 g OGTT	24	23	53
Glucose level at 2 h during the 75 g OGTT	7	14	79
Glucose level at 3 h during the 75 g OGTT	58	28	14
Insulin level at 30 min during the OGTT	53	31	16
Insulin level at 2 h during the OGTT	39	37	24
Fasting glucose	3	15	82
Random glucose	45	36	19
HbA_1c_ level	4	13	83
Fasting HbA_1c_	50	20	30
Insulin level	38	34	28
Fasting insulin level	28	36	36
Insulin to glucose ratio	28	40	32
Insulinogenic index	30	42	28
Corrected insulin response	37	40	23
Insulin sensitivity	25	41	33
HOMA	29	42	29
HOMA-IR	26	39	35
HOMA-β	28	40	32
Triacylglycerol level	24	36	40
Fasting triacylglycerol level	12	33	55
Cholesterol level	21	37	42
Fasting cholesterol	14	37	49
HDL	20	36	44
Fasting HDL	15	38	47
LDL	20	33	47
Fasting LDL	16	36	48
Apo-lipoprotein B	35	46	19
Oxidised lipoproteins	38	46	16
ACR	11	37	52
Insulin growth factor	45	39	16
IGF-binding protein 3 level	50	37	13
Paraoxonase	54	33	13
Leptin	40	40	20
Adiponectin	39	42	19
Total circulating adiponectin	43	42	15
High sensitivity C-reactive protein	35	45	20
Fibrinogen	47	39	14
Alanine aminotransferase	23	46	31
Thyroid stimulating hormone	16	40	44
Free thyroxine	28	35	37
Total tissue plasminogen activator antigen	49	39	12
Cytokines	47	41	12
Genotype for Pro12Ala polymorphism	57	33	10
Plasma uric acid	41	38	21
γ-glutamyl aminotransferase	30	43	27
Vitamin D	26	42	32
Clinical conditions			
Type 2 diabetes	0	3	97
Insulin-treated type 2 diabetes	3	8	89
GDM in subsequent pregnancies	0	4	96
The metabolic syndrome	2	16	82
Impaired fasting glucose	2	14	84
Impaired glucose tolerance	2	14	84
Polycystic ovary syndrome	4	26	70
Cardiovascular disease	2	17	81
Physiological variables			
Weight	1	4	95
Height	12	12	76
BMI	1	13	86
Waist circumference	2	18	80
Hip circumference	9	34	57
Waist/hip ratio	7	28	65
Body fat	8	37	55
Lean mass	7	45	48
Post-pregnancy weight retention	2	24	74
BP	1	11	88
Resting BP	2	15	83
Systolic BP	2	12	86
Diastolic BP	2	12	86
Resting pulse	6	38	56
Diet and exercise			
Coffee intake	22	56	22
Tea intake	25	55	20
Alcohol intake	5	32	63
Protein intake	12	45	43
Carbohydrate intake	8	27	65
Fruit intake	10	28	62
Vegetable intake	9	29	62
Dairy intake	8	39	52
Fat intake	10	32	58
Fibre intake	9	38	53
Low glycaemic intake	10	35	55
Unsaturated fat intake	13	32	55
Saturated fat intake	12	31	57
Healthy fat intake	14	29	57
Low fat intake	18	40	42
Low carbohydrate high-fat diet	17	37	46
Nutrition composition	13	34	53
Food frequency	13	29	58
Portion size	7	27	66
Total caloric intake	7	26	67
Sweetened beverage intake	9	23	68
Physical activity	2	9	89
Walking/cycling to work	7	23	70
Time spent sitting	6	23	71
Time spent sleeping	9	26	65
Psychological variables			
Quality of life	3	18	79
Eating behaviour	6	24	70
Adverse emotional status	4	28	68
Mental health status	4	23	73
Anxiety	4	30	66
Depression	4	23	73
Activity to enhance mood	8	28	64
Postnatal depression	5	15	80
Other			
Smoking status	4	7	89
Quality adjusted life year	6	30	64
Healthcare costs	11	30	59
Lost productivity due to absence from work	11	39	50
Sickness resulting in absence from work	11	37	52
Satisfaction with research participation	15	41	44
Number of pregnancies since the index pregnancy	6	17	77
Health insurance	25	38	37
Income	20	40	40
Menopause status	17	35	48
Miscarriage after the index pregnancy	11	29	60
Breastfeeding in the index pregnancy	5	16	79
Breastfeeding at 1 year post delivery	10	28	62
Plans for future pregnancies	10	24	66
Employment status	21	35	44
Return to work after pregnancy	18	41	41
Current medications	5	21	74
Oral contraceptive use	14	23	63
Contraception method	16	27	57
Hormone replacement therapy	16	30	44
Hysterectomy	25	35	40
Oophorectomy	26	37	47
Genital malignancies	20	30	40

Round 3 was completed by 165 participants (86.8%). Similar to round 2, outcomes were brought forward when 70% or more participants scored the outcome as 7–9 and <15% participants scoring as 1–3. In total, 30 outcomes went through the consensus meeting (ESM Table 3).

### Consensus meeting

The consensus meeting involved 20 participants, a chairperson and an administrator. The stakeholder groups included four women with a history of gestational diabetes, one diabetes nurse specialist, two midwives, one policy maker, two paediatricians, one clinical biochemist, two researchers in the area of diabetes in pregnancy, one epidemiologist, two obstetricians and four endocrinologists. The participants represented ten countries and three continents. Before the discussion on each outcome, the participants were shown the previous voting results on that particular outcome by the total group and by each stakeholder group. Each outcome was discussed and there was agreement that some items should be grouped and/or rephrased. Therefore, ‘75 g oral glucose tolerance test’, ‘Blood glucose level at 2 h during the 75 g oral glucose tolerance test’, ‘Fasting glucose’ and ‘HbA_1c_ blood levels’ were combined into ‘Assessment of glycaemic status’. ‘Type 2 diabetes’ became ‘Diagnosis of type 2 diabetes since the index pregnancy’. ‘GDM in subsequent/future pregnancies’ became ‘Number of pregnancies with a diagnosis of GDM since the index pregnancy’. ‘Impaired fasting glucose’ and ‘Impaired glucose tolerance’ were combined into ‘Diagnosis of prediabetes since the index pregnancy’ and ‘Breastfeeding after the index pregnancy’ became ‘Breastfeeding’.

Following discussion, the panel voted on each outcome to determine whether it should or should not be included in the final set. The final COS included nine outcomes and is presented in Table 3, together with the percentage of participants that voted ‘consensus in’.

**Table 3:** Final outcomes to be included in the COS

Outcome	Votes for the outcome to be included in the final COS, %
Assessment of glycaemic status	100
Diagnosis of T2DM since the index pregnancy	90
Number of pregnancies since the index pregnancy	100
Number of pregnancies with a diagnosis of GDM since the index pregnancy	100
Diagnosis of prediabetes since the index pregnancy	100
BMI	100
Post-pregnancy weight retention	95
Resting BP	100
Breastfeeding	75

T2DM, type 2 diabetes mellitus

## Discussion

This study used robust methods to develop the first COS relevant to the follow-up of women with previous gestational diabetes treated with insulin and/or glucose-lowering agents. A Delphi consensus panel with 20 representatives from ten countries agreed on nine outcomes to be included in the final COS. It is advised that all studies in this area use this COS to facilitate comparison among studies and limit heterogeneity and reporting bias. The application of agreed methods in developing a COS and the participation of multiple stakeholder groups assure the wide applicability and dissemination of this COS.

The wide applicability of the study was one of the main reasons why the outcomes ‘75 g oral glucose tolerance test’, ‘Blood glucose level at 2 h during the 75 g oral glucose tolerance test’, ‘Fasting glucose’ and ‘HbA_1c_ blood levels’ were combined into ‘Assessment of glycaemic status’; in so doing, the COS permits researchers the opportunity to assess glycaemic status according to their own national guidelines and resources. In addition, this COS identifies ‘what is to be collected’ and not ‘how it is to be collected’, which will be the subject of future work. Similarly, ‘Impaired fasting glucose’ and ‘Impaired glucose tolerance’ were combined as ‘Diagnosis of prediabetes since the index pregnancy’ to give the COS a worldwide applicability in light of the variability in diagnostic tools and criteria. However, while we would recommend that collection and reporting of all outcomes in the COS is mandatory, researchers can choose to collect any additional outcomes required for their study, including specific indices of glycaemic control.

Of importance, the COS consensus meeting had representation from a variety of health professionals/specialties, both local and international, and included women with a previous diagnosis of GDM. This stakeholder composition permitted both the health professionals and women to bring their experience and perspectives to the issues under discussion. Each participant was able to have an understanding of what was important to the other person and what was feasible. Ultimately, this resulted in shared decision-making in a study that will impact future research of women with gestational diabetes.

There are some limitations to our study. Currently, there are no methods for sample size calculation for this type of study. To minimise the potential for selection bias, participants were invited through international organisations and personal professional email lists. Only participants who completed each survey round were recorded. From the totality of initial emails sent before round 1, we had a 35% response. This response rate, however, included participants from 33 countries and five continents. We had a 34% drop-off rate between round 1 and round 2, but, impressively, 87% of participants who completed round 2 also completed round 3. There was a low response rate among primary care physicians (3.5%).

Another potential limitation of this study is the large number of items to be scored in rounds 1 and 2 of the online Delphi survey, which may have impacted negatively on the survey response rate.

We had a large percentage of international participants, including individuals from high-income, upper- and lower-middle-income countries. However, low-income countries were under-represented, and this may limit the generalisability of this COS to certain parts of the world. The participants at the consensus meeting tried to overcome this by rephrasing or combining outcomes in order to increase the worldwide applicability of the COS.

The access to service users was limited by data protection laws, so participants were recruited through the clinical facilities. Despite this, 30% of respondents in round 1 were international service users. At the consensus meeting, 50% of service user participants were non-Irish. We sought international participation for the COS to have global relevance. The service user representatives made important suggestions on all the outcomes discussed at the consensus meeting. Anonymous voting (via the electronic app during this meeting) aimed to prevent participants feeling compelled to vote in a certain way. It has been advocated that the views of health service users should be given greater value in the development of a COS [20] as outcomes reported for clinical studies might not reflect endpoints that are meaningful for them. Examples exist where health service users identified an outcome important to them as a group that might not have been considered by clinicians [21–23]. However, the responses from health service users and the responses from other stakeholder groups were generally concordant.

The strengths of the study include our use of robust methods in the COS development, including adherence to the COS-STAR statement, the thoroughness of the systematic review (six databases were searched for relevant studies), the high number of participants and the diversity of stakeholder groups participating at each stage of the COS.

Recent review papers have shown the degree of outcome reporting bias among studies, with the main outcome not being reported in up to 47% of the studies and inadequately reported in up to 76% of the studies reviewed [24–26]. Therefore, there is a cogent argument for creating and disseminating a COS to harmonise outcome reporting.

This is the first study to outline a COS for the long-term follow-up of women with previous GDM. We encourage all investigators undertaking research in this field to report, as a minimum, this COS to reduce reporting bias by allowing evidence synthesis across clinical studies. This will ultimately lead to improvements in the quality of research and delivery of evidence-based healthcare for women with GDM.

## Electronic supplementary material

ESM (PDF 281 kb)

## Data Availability

Data are available on request.

## Abbreviations

COMET: Core Outcome Measures in Effectiveness Trials Initiative
COS: Core outcome set
COS-STAR: Core Outcome Set STAndards for Reporting
GDM: Gestational diabetes mellitus
IADPSG: International Association of Diabetes in Pregnancy Study Group
SAG: Study advisory group
