# Supplementary material for: Follow-up at 1 year and beyond of women with gestational diabetes treated with insulin and/or oral glucose-lowering agents: a core outcome set using a Delphi survey
Source: Diabetologia. 2019 Jul 4;62(11):2007–16. doi: 10.1007/s00125-019-4935-9 (PMC6805965; doi:10.1007/s00125-019-4935-9)

ESM Methods

Search strategy PubMed

1. Gestational diabetes
2. Gestational diabetes OR GDM OR diabetes in pregnancy
3. Insulin
4. 2 AND 3
5. 4 AND follow-up
6. 4 AND outcome
7. 4 AND outcomes
8. 4 AND follow
9. 5 OR 6 OR 7 OR 8
10. 4 AND postpartum
11. 4 AND post partum
12. 4 AND post-partum
13. 9 OR 10 OR 11 OR 12
14. 2 AND treatment
15. 2 AND follow-up
16. 2 AND Oral
17. 2 AND Oral hypoglycemic
18. 2 AND Oral hypoglycaemic
19. 2 AND Oral antihyperglycemic
20. 2 AND Oral antihypergycaemic
21. 2 AND metformin
22. 2 AND medication
23. 2 AND glyburide
24. 2 AND pharmacological
25. 2 AND antidiabetic
26. 2 AND anti-diabetic
27. 2 AND anti diabetic
28. 2 AND acarbose
29. 4 OR 14 OR 16 OR 17 OR 18 OR 19 OR 20 OR 21 OR 22 OR 23 OR 24 OR 25 OR 26 OR 27 OR 28
30. 29 AND follow
31. 29 AND follow-up
32. 29 AND postpartum
33. 29 AND post-partum
34. 29 AND post partum
35. 29 AND future
36. 29 AND risk
37. 35 OR 36
38. 32 OR 33 OR 34
39. 30 OR 31
40. 37 OR 38 OR 39

(plus additional filters provided by the website on the type of study)

ESM Table 1. Papers eligible for full text review

**10-year follow-up of diabetes incidence and weight loss in the Diabetes Prevention Program Outcomes Study.**<br>**Diabetes Prevention Program Research Group, Knowler WC, Fowler SE, Hamman RF, Christophi CA, Hoffman HJ, Brenneman AT, Brown-Friday JO, Goldberg R, Venditti E, Nathan DM.**<br>**Lancet. 2009 Nov 14;374(9702):1677-86. doi: 10.1016/S0140-6736(09)61457-4. Epub 2009 Oct 29.**
**A Pregnancy and Postpartum Lifestyle Intervention in Women With Gestational Diabetes Mellitus Reduces Diabetes Risk Factors**<br>**Assiamira Ferrara, MD, PHD, Monique M. Hedderson, PHD, Cheryl L. Albright, PHD, MPH,Samantha F. Ehrlich, MPH, Charles P. Quesenberry, Jr., PHD, Tiffany Peng, MA, Juanran Feng, MS,Jenny Ching, RN, andYvonne Crites, MD**<br>**Diabetes Care. 2011 Jul; 34(7): 1519–1525.Published online 2011 Jun 17. doi: 10.2337/dc10-2221**
**A Randomised Controlled Trial to Delay or Prevent Type 2 Diabetes after Gestational Diabetes: Walking for Exercise and Nutrition to Prevent Diabetes for You**<br>**S. Peacock, F. E. Bogossian,S. A. Wilkinson,K. S. Gibbons, C. Kim,and H. D. McIntyre**<br>**International Journal of Endocrinology, Volume 2015 (2015), Article ID 423717, 8 pages, http://dx.doi.org/10.1155/2015/423717**
**A web-based lifestyle intervention for women with recent gestational diabetes mellitus: a randomized controlled trial.**<br>**Nicklas JM, Zera CA, England LJ, Rosner BA, Horton E, Levkoff SE, Seely EW.**<br>**Obstet Gynecol. 2014 Sep;124(3):563-70. doi: 10.1097/AOG.0000000000000420.**

**Adiponectin, C- reactive protein, fibrinogen and tissue plasminogen activator antigen levels among glucose-intolerant women with and without histories of gestational diabetes.**<br>**Kim C Christophi CA, Goldberg RB, Perreault L, Dabelea D, Marcovina SM, Pi-Sunyer X, Barrett-Connor E.**<br>**Diabet Med. 2016 Jan;33(1):32-8. doi: 10.1111/dme.12799. Epub 2015 May 29.**
**Changes in Insulin Sensitivity in Response to Troglitazone Do Not Differ Between Subjects With and Without the Common, Functional Pro12Ala Peroxisome Proliferator-Activated Receptorγ2 Gene Variant.**<br>**Soren Snitker, MD,PHD, Richard M. Watanabe, PHD, Ifeanyi Ani, BA, Anny H. Xiang, PHD, Aura Marroquin, RN, BSN, Cesar Ochoa, MD, Jose Goico, MD, Alan R. Shuldiner, MD, and Thomas A. Buchanan, MD**<br>**Diabetes Care. 2004 Jun; 27(6): 1365–1368.**
**Comparison of Two Screening Strategies for Gestational Diabetes (GDM^2) Trial: Design and rationale**<br>**Kaleab Z. Abebe, Christina Scifres, Hyagriv N. Simhan, Nancy Day, Patrick Catalano, Lisa M. Bodnari, Tina Costacou, Deanna Matthew, Alexandra Illes, Steven Orris, Jessica Duell, Kathleen Ly, Esa M. Davis**<br>**Contemporany Clinical Trials, November 2017, Volume 62, Pages 43-49 DOI: https://doi.org/10.1016/j.cct.2017.08.012**
**Diabetes mellitus and abnormal glucose tolerance development after gestational diabetes: A three-year, prospective, randomized, clinicalbased, Mediterranean lifestyle interventional study with parallel groups**<br>**Natalia Perez-Ferre , Laura Del Valle , Maria Jose Torrei, Idoya Barca , María Isabel Calvo, Pilar Matía , Miguel A. Rubio , Alfonso L. Calle-Pascual**

**Clinical Nutrition, August 2015, volume 34, issue 4, pages 579-585**
**Do postal reminders increase postpartum screening of diabetes mellitus in women with gestational diabetes mellitus? A randomized controlled trial.**<br>**Clark HD, Graham ID, Karovitch A, Keely EJ.**<br>**Am J Obstet Gynecol. 2009 Jun;200(6):634.e1-7. doi: 10.1016/j.ajog.2009.01.003. Epub 2009 Mar 9.**
**Effectiveness and Cost-Effectiveness of a Cluster-Randomized Prenatal Lifestyle Counseling Trial: A Seven-Year Follow-Up; Päivi Kolu, Jani Raitanen, Jatta Puhkala,Pipsa Tuominen,Pauliina Husu, and Riitta Luoto; PLoS One. 2016; 11(12): e0167759.**<br>**Published online 2016 Dec 9. doi: 10.1371/journal.pone.0167759**
**Genetic Risk of Progression to Type 2 Diabetes and Response to Intensive Lifestyle or Metformin in Prediabetic Women With and Without a History of Gestational Diabetes Mellitus**<br>**Shannon D. Sullivan, Kathleen A. Jablonski, Jose C. Florez,Dana Dabelea, Paul W. Franks, Sam Dagogo-Jack, Catherine Kim, William C. Knowler, Costas A. Christophi, Robert Ratner, and for the Diabetes Prevention Program Research Group**<br>**Diabetes Care. 2014 Apr; 37(4): 909–911.Published online 2014 Mar 8. doi: 10.2337/dc13-0700**
**Menopause and the risk of diabetes in the diabetes prevention program**<br>**Catherine Kim, M.D., M.P.H., Sharon L. Edelstein, Sc.M., Jill P. Crandall, M.D., Dana Dabelea, M.D., Ph.D., Abbas E. Kitabchi, Ph.D., M.D., Richard F. Hamman, M.D., Dr.Ph., Maria G. Montez, R.N., M.S.H.P., Leigh Perreault, M.D., Mary A. Foulkes, Ph.D., and Elizabeth Barrett-Connor, M.D., for the Diabetes Prevention Program Research Group**

**Menopause. 2011 Aug; 18(8): 857–868. doi: 10.1097/gme.0b013e31820f62d0**
**Post-partum weight loss and glucose metabolism in women with gestational diabetes: the DEBI Study**<br>**S. F. Ehrlich, M. M. Hedderson, C. P. Quesenberry Jr, J. Feng, S. D. Brown, Y. Crites, and A. Ferrara**<br>**Diabet Med. 2014 July ; 31(7): 862–867. doi:10.1111/dme.12425.**
**Pregnancy-Associated Hypertension in Glucose Intolerant Pregnancy and Subsequent Metabolic Syndrome**<br>**Madeline Murguia Rice, Ph.D., Mark B. Landon, M.D., Michael W. Varner, M.D., Brian M. Casey, M.D., Uma M. Reddy, M.D., M.P.H., Ronald J. Wapner, M.D., Dwight J. Rouse, M.D., Joseph R. Biggio, Jr., M.D., John M. Thorp, Jr., M.D., Edward K. Chien, M.D., M.B.A., George Saade, M.D., Alan M. Peaceman, M.D., Sean C. Blackwell, M.D., and J. Peter VanDorsten, M.D.**<br>**Obstet Gynecol. 2016 Apr; 127(4): 771–779. doi: 10.1097/AOG.0000000000001353**
**Preservation of pancreatic beta-cell function and prevention of type 2 diabetes by pharmacological treatment of insulin resistance in high-risk Hispanic women.**<br>**Buchanan TA, Xiang AH, Peters RK, Kjos SL, Marroquin A, Goico J, Ochoa C, Tan S, Berkowitz K, Hodis HN, Azen SP.**<br>**Diabetes. 2002 Sep;51(9):2796-803.**

**Prevalence of metabolic markers of insulin resistance in offspring of gestational diabetes pregnancies**<br>**Erin J Keely, Janine C Malcolm, Stasia Hadjiyannakis, Isabelle Gaboury , Gigi Lough ,Margaret L Lawson**<br>**Pediatr Diabetes. 2008 Feb;9(1):53-9. Epub 2007 Nov 23.**
**Prevention of diabetes in women with a history of gestational diabetes: effects of metformin and lifestyle interventions**<br>**Robert E. Ratner Costas A. Christophi Boyd E. Metzger Dana Dabelea Peter H. BennettXavier Pi-Sunyer Sarah Fowler Steven E. KahnThe Diabetes Prevention Program Research Group**<br>***The Journal of Clinical Endocrinology & Metabolism*, Volume 93, Issue 12, 1 December 2008, Pages 4774–4779, https://doi.org/10.1210/jc.2008-0772**
**Prevention of gestational diabetes through lifestyle intervention: study design and methods of a Finnish randomized controlled multicenter trial (RADIEL).**<br>**Rönö K, Stach-Lempinen B, Klemetti MM, Kaaja RJ, Pöyhönen-Alho M, Eriksson JG, Koivusalo SB; RADIEL group.**<br>**BMC Pregnancy Childbirth. 2014 Feb 14;14:70. doi: 10.1186/1471-2393-14-70.**
**Prevention of Gestational Diabetes: Design of a Cluster-Randomized Controlled Trial and One-Year Follow-Up**<br>**Riitta M Luoto Tarja I Kinnunen, Minna Aittasalo, Katriina Ojala, Kirsi Mansikkamäki, Erja Toropainen, Päivi Kolu and Tommi Vasankari**<br>**BMC Pregnancy and Childbirth201010:39https://doi.org/10.1186/1471-2393-10-39**
**Postpartum glucose intolerance in Chinese women with gestational diabetes**<br>**X. Yang, BH.-H. Hsu-Hage, L. Dong, H. Zhang, C. Zhang and Y. Zhang**<br>**Diabetes UK. Diabetic Medicine,2003, 20, 686–690**

**Reasons for participation and non-participation in a diabetes prevention trial among women with prior gestational diabetes mellitus (GDM)**<br>**Jennifer J Infanti, Angela O’Dea,Irene Gibson, Brian E McGuire, John Newell,Liam G Glynn,Ciaran O’Neill,Susan B Connolly, and Fidelma P Dunne**<br>**BMC Med Res Methodol. 2014; 14: 13.**<br>**Published online 2014 Jan 24. doi: 10.1186/1471-2288-14-13**
**Screening for type 2 diabetes following gestational diabetes. Family phisicians and patients perspective;**<br>**Erin Keely, Heather Clark, Alan Karovitch, Ian Graham**<br>**Can Fam Physician. 2010 Jun; 56(6): 558–563.**
**The effect of lifestyle intervention and metformin on preventing or delaying diabetes among women with and without gestational diabetes: the Dabetes Prevention Program outcomes study 10-year follow-up.**<br>**Aroda VR, Christophi CA, Edelstein SL, Zhang P, Herman WH, Barrett-Connor E, Delahanty LM, Montez MG, Ackermann RT, Zhuo X, Knowler WC, Ratner RE; Diabetes Prevention Program Research Group.**<br>**J Clin Endocrinol Metab. 2015 Apr;100(4):1646-53. doi: 10.1210/jc.2014-3761. Epub 2015 Feb 23.**
**The effects of metformin treatment of gestational diabetes on maternal weight ad glucose tolerance postpartum – a prospective follow-up study.**<br>**Pellonperä O, Rönnemaa T, Ekblad U, Vahlberg T, Tertti K.**<br>**Acta Obstet Gynecol Scand. 2016 Jan;95(1):79-87. doi: 10.1111/aogs.12788. Epub 2015 Nov 8.**
**Tiajin gestational diabetes prevention program**

Gang Hu, Huiguang Tian, Fuxia Zhang, Huikun Liu, Cuiping Zhang, Shuang Zhang, Leishen Wang. Gongshu Liu, Zhijie Yu, Xilin Yang, Lu Qi, Cuilin Zhang, Hua Wang, Min Li, Junhong Leng, Yi Li, Ling Dong, Jaakko Tuomilehto

Diabetes Research and Clinical Practice, December 2012, volume 98, issue 3, pages 508-517

ESM Table 2. Additional outcomes to be included in round 2

**1. Developing gestational diabetes in subsequent pregnancies**
**2. Developing a genital tract malignancy**
**3. Developing cardiovascular disease**
**4. Breastfeeding at one-year post delivery**
**5. Vitamin D level**
**6. Post-pregnancy weight retention**
**7. Current diabetes treated with insulin**
**8. Adopting a low carbohydrate high fat diet**
**9. Decision regarding future pregnancy plans**
**10. Contraception method**

ESM Table 3. Outcomes going through the consensus meeting following round 3 of voting

**75 g Oral Glucose Tolerance Test**
**Blood glucose level at 2 h during the 75 g Oral Glucose Tolerance Test**
**Fasting glucose**
**HbA1c blood levels**
**Type 2 diabetes**
**Insulin treated type 2 diabetes**
**Gestational diabetes in subsequent/future pregnancies**
**Metabolic syndrome**
**Impaired fasting glucose**
**Impaired glucose tolerance**
**Polycystic ovary syndrome**
**Cardiovascular disease**
**Weight**
**Height**

**Body Mass Index**
**Waist circumference**
**Post-pregnancy weight retention**
**Blood pressure**
**Resting blood pressure**
**Systolic blood pressure**
**Diastolic blood pressure**
**Physical activity**
**Walking/cycling to work**
**Time spent sitting**
**Quality of life**
**Eating behaviour**
**Mental health status**
**Depression**
**Post-natal depression**

**Smoking status**
**Number of pregnancies since the index pregnancy**
**Breastfeeding after the index pregnancy**
**Current medications**

ESM Fig.1 Selection of studies for systematic review

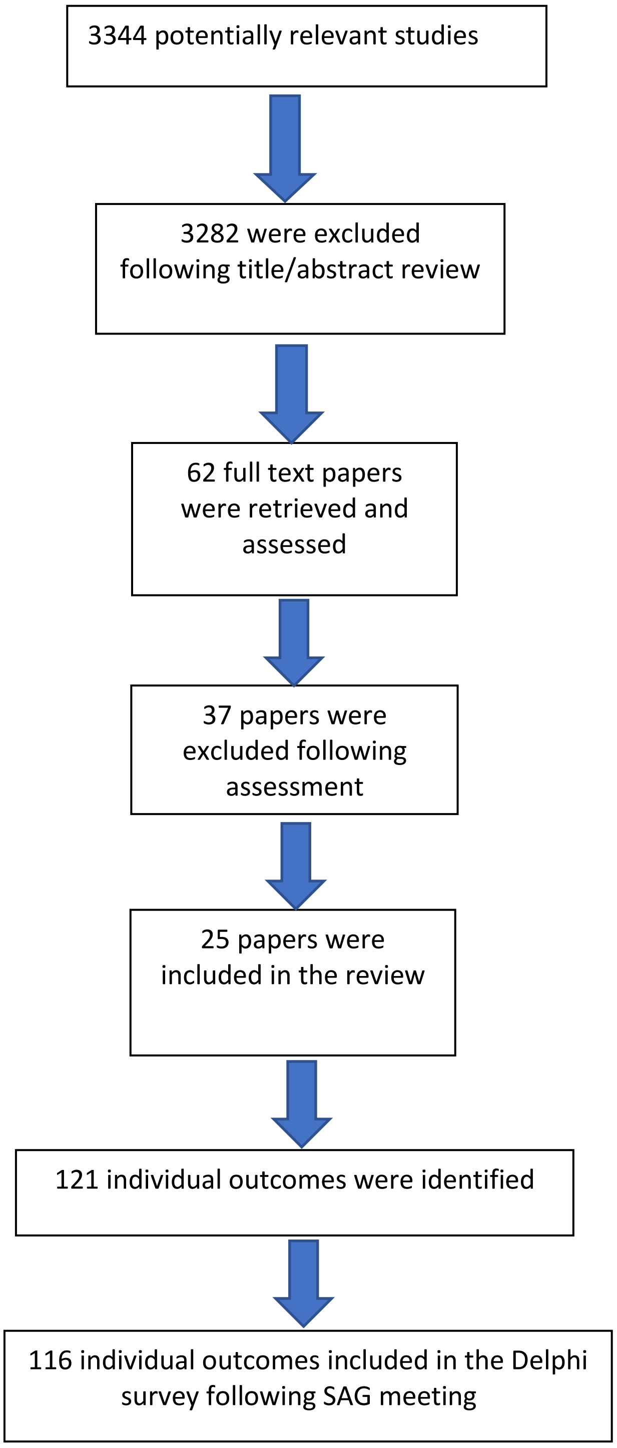

Supplement: Supplementary file 1 — (PDF 281 kb) [file 125_2019_4935_MOESM1_ESM.pdf]
